# Modeling High-Grade Serous Carcinoma: How Converging Insights into Pathogenesis and Genetics are Driving Better Experimental Platforms

**DOI:** 10.3389/fonc.2013.00217

**Published:** 2013-08-26

**Authors:** Paul Michael Jones, Ronny Drapkin

**Affiliations:** ^1^Department of Medical Oncology, Harvard Medical School, Dana-Farber Cancer Institute, Boston, MA, USA

**Keywords:** ovarian cancer, genetics, pathogenesis, model systems, OSE, fallopian tube

## Abstract

Recent developments in the study of epithelial ovarian cancer have called into question the traditional views regarding the site of tumor initiation. Histopathologic studies and genomic analyses suggest that extra-ovarian sites, like the fallopian tube, may harbor the coveted cell of origin and could therefore contribute significantly to the development of high-grade serous ovarian carcinoma (HG-SOC). Our ability to validate these emerging genomic and pathologic observations and characterize the early transformation events of HG-SOC hinges on the development of novel model systems. Currently, there are only a handful of new model systems that are addressing these concerns. This review will chronicle the convergent evolution of these ovarian cancer model systems in the context of the changing pathologic and genomic understanding of HG-SOC.

## Introduction

In 2013, the American Cancer Society estimates that 22,240 women will receive a new diagnosis of ovarian cancer and that 14,030 women will die from this disease, making ovarian cancer the most lethal gynecological malignancy in the United States (1). Of these newly diagnosed cases, 80% of the serous ovarian carcinomas are diagnosed at late stage, for which the 5-year survival rate is only 9–35% (2). Despite advancements in technology, this poor survival rate has been consistent over the last 30 years, an indictment of the complexity of this disease. In order to combat this clinical challenge, it is imperative to generate robust early detection methods and novel treatment options.

**Figure 1:**
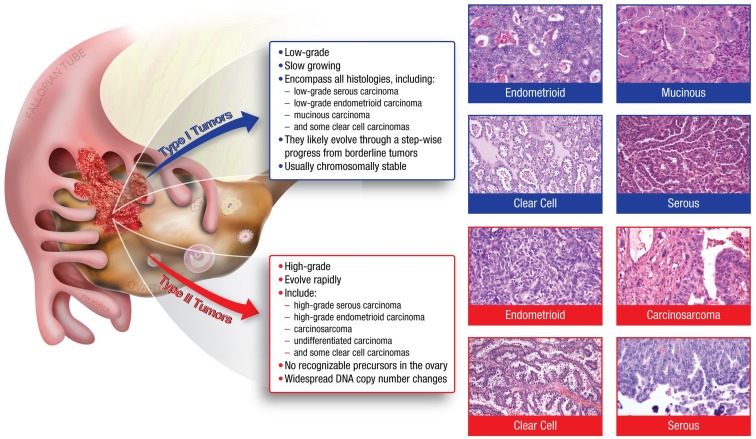
**The major histologic subtypes of ovarian cancer fall into two subclassifications**. Type I tumors are low-grade, slow growing carcinomas that typically arise from well recognized precursors lesions (borderline tumors) that themselves develop from the ovarian surface epithelium, inclusion cysts, or endometriosis. In contrast, Type II tumors are high-grade and rapidly growing carcinomas. Typically, they have spread well beyond the ovary at the time of diagnosis.

Many of the characteristics confounding the study of ovarian cancer arise from the disease’s heterogeneity. Ovarian tumors can arise from three different cell types; epithelial, germ, and sex cord stromal cells, with epithelial accounting for approximately 90% of all ovarian cancers (1). Epithelial tumors are further grouped into different tumor types: Type I and Type II. Type I tumors include low-grade serous carcinoma, low-grade endometrioid carcinoma, mucinous carcinoma, and a subset of clear cell carcinomas, which develop in a stepwise fashion from well-recognized precursors, in most cases, borderline tumors (3–5) (Figure 1). These tumors are slow to develop and are generally confined to the ovary (6). Type I tumors are also genetically stable, with each histologic subtype corresponding to a distinct genetic profile (4–6). In contrast, Type II tumors encompass high-grade serous carcinoma, undifferentiated carcinoma, malignant mixed mesodermal tumor (carcinosarcoma), and some clear cell carcinomas (3) (Figure 1). High-grade serous carcinomas are the most common Type II tumor. These tumors progress rapidly, harbor *TP53* mutations, and exhibit widespread DNA copy number alterations (3–7).

This new appreciation of tumor diversity and the rapid development of genomic technologies have helped redefine “ovarian cancer.” As the field grapples with these emerging concepts, experimental model systems will likely play a vital role in defining new opportunities for early detection and therapeutic intervention. This review will highlight the recent advancements in ovarian cancer genetics and pathology, and explore the past and present model systems employed to study high-grade serous ovarian carcinoma (HG-SOC).

## Genetics

Until recently, neoplastic transformation was thought to be driven by the sequential acquisition of mutations in critical genes. For many epithelial cancers, including Type I ovarian cancer, this is true. The most prominent mutations present in Type I tumors include alterations to *KRAS, BRAF, PTEN*, *CTNNB1*, and *TGFBR2* (3, 6, 8). However, besides mutations in the *TP53* tumor suppressor gene and the *BRCA1* or *BRCA2* genes, very few recurrent somatic mutations have been associated with the more aggressive Type II tumors (6). This inability to systematically characterize Type II tumors was addressed by the National Cancer Institute (NCI) and the National Human Genome Research Institute (NHGRI) in the creation of The Cancer Genome Atlas (TCGA). In the TCGA’s pilot study of HG-SOC, microarray analyses and new sequencing technology were used to publish the largest and most comprehensive genetic analysis of HG-SOC. The study encompassed mRNA expression, microRNA expression, DNA copy number, and DNA promoter region methylation for 489 HG-SOC and whole exome DNA sequence information for 316 of these samples (7).

Results from the initial TCGA study characterized HG-SOC as having *TP53* mutations in nearly 100% of tumors and identified low prevalence but statistically significant recurrent somatic mutations in nine additional genes including *NF1, BRCA1, BRCA2, RB1*, and *CDK12* (7). TCGA also described 113 DNA copy number alterations and implicated 168 genes involved in promoter methylation events (7). Considering the widespread DNA copy number aberrations observed across HG-SOC, it has been suggested that disruption of DNA repair pathways followed by chromosome instability is a viable model for the early progression of HG-SOC (9, 10). The TCGA provides an expanding database that is useful in identify high impact genes. However, because the TCGA studies the advanced state of HG-SOC, determining whether these genes are important to transformation, or instead are related to tumor maintenance, immune evasion, anti-apoptosis, and/or chemoresistance, requires further investigation.

## Pathogenesis

Historically, ovarian cancer was believed to originate from the ovarian surface epithelium (OSE), where ovulation, follicular rupture, oocyte release, cytokine exposure, and reactive oxygen species introduce DNA damage into the ovarian epithelial layer (11, 12). Proposed back in 1971, the Fathalla “incessant ovulation” hypothesis (13) suggests that over a woman’s lifespan, the accrual of DNA damage and the development of cortical inclusion cysts (CICs) results in Mullerian metaplasia of the coelomic epithelium followed by neoplastic transformation (14, 15). This hypothesis attempts to explain the presentation of coexisting serous and non-serous tumor subtypes within ovarian tumors and incorporates the epidemiological data linking ovulatory activity with risk of ovarian cancer (16). However, while precursor lesions have been identified in the OSE that are linked to Type I tumors (17), reproducible pre-malignant lesions have been difficult to identify in the OSE for the high-grade Type II tumors.

A more recent analysis compares the major subtypes of ovarian carcinomas to tumors arising in the fallopian tube, endometrium, and endocervix. Evidence suggests that benign structures derived from these anatomic locations may serve as sites of origin for all tumors that have traditionally been regarded as of primary ovarian origin. Such epithelial structures, which include endosalpingiosis, endometriosis, and endocervicosis, represent non-neoplastic counterparts of serous, endometrioid/clear cell, and mucinous ovarian carcinomas, respectively, and are referred to as extra-uterine Müllerian epithelium (EUME) (15).

**Figure 2:**
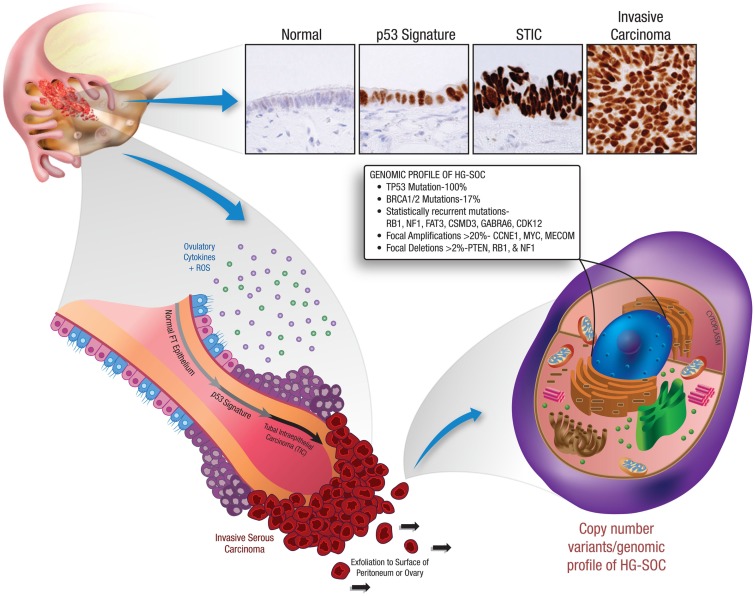
**Early tumor progression within the fallopian tube and the resultant genetic profile of HG-SOC**. This illustration depicts the recently identified precursor lesions of HG-SOC that are present in the fallopian tube. Mutations in the *TP53* tumor suppressor gene are a very early event in the pathogenesis of HG-SOC, occurring exclusively in benign-appearing secretory cells. These preneoplastic lesions are referred to as ‘p53 signatures’. Acquisition of a neoplastic phenotype and proliferative capacity results in the development of serous tubal intraepithelial carcinoma (STIC). Breaching of the basement membrane and localized dissemination to the ovary and/or peritoneal cavity heralds the development of invasive HG-SOC and the associated clinical scenario. HG-SOCs that involve the ovary or peritoneum are characterized by mutations in *TP53* (and *BRCA1* in familial cases) and display a complex genomic terrain with widespread copy number alterations throughout the genome.

The most significant studies supporting the concept of EUME are those implicating the fallopian tube fimbria as the site of origin for high-grade serous carcinomas. Early studies of fallopian tube carcinomas noted *TP53* and *interleukin 6* (*IL-6*) mutations (18, 19). However, a link to ovarian cancer was not proposed until pathologists systematically analyzed fallopian tubes from women carrying mutations in the *BRCA1* tumor suppressor gene. These studies identified preneoplastic lesions localized to the tubal fimbria (20–22), where they displayed secretory cell histology, DNA damage, mutations in *TP53*, and stable p53 protein expression (20, 23). This evidence suggests that HG-SOC tumor progression within the fallopian tube fimbria begins with *TP53* mutations (*p53* signatures), evolves to serous tubal intraepithelial carcinoma (STIC), and eventually transforms and metastasizes to the ovary presenting as HG-SOC (20, 24) (Figure 2).

## Experimental Models of HG-SOC

**Figure 3:**
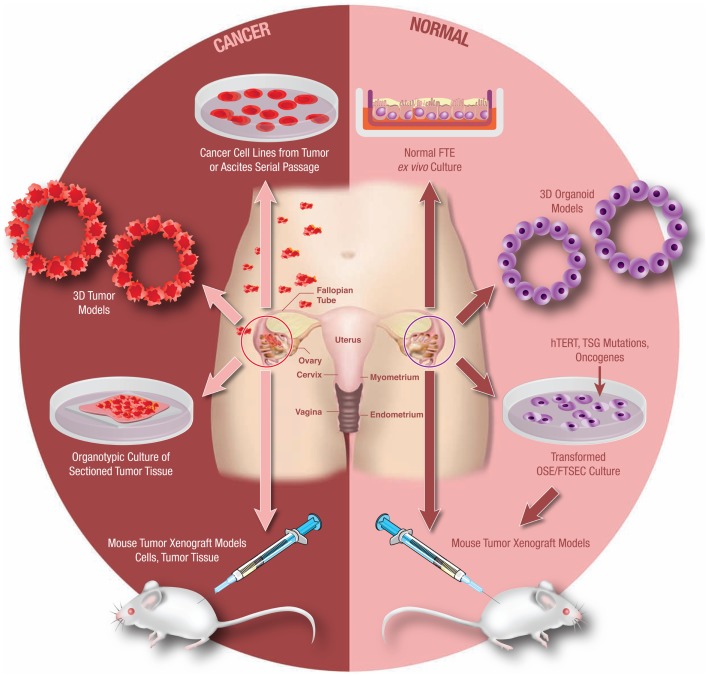
**Model systems from primary human tissues**. An array of experimental model systems spanning *in vitro* and *in vivo* approaches have been developed to study ovarian cancer. The models include platforms to interrogate the biology of cancer cells as well as for the study of benign epithelium. The expansion beyond traditional two-dimensional (2D) cell culture into 3D and organoid cultures has yielded important insights into the biology of this disease, as has the development of unique animal models. Development of these models is critical as our understanding of this cancer continues to evolve.

Experimental models systems in ovarian cancer biology have evolved significantly over the past 10–15 years. Today there exist a number of useful models that continue to advance translational research in ovarian cancer (Figure 3). It is beyond the scope of this mini-review to address all available experimental models. However, in order to demonstrate the utility and evolution of these research tools, a select few of these models will be discussed.

### Xenografts

Xenograft models are possibly the most utilized experimental platform in the field of cancer research. Early developments of this model were reported in the late 1960s when Rygaard et al. found that mice suffering from recessive thymic aplasia could grow mammary and colon xenografted carcinomas (25). This effectively spawned the immunocompromised rodent model, which, due to its ease of application and histological insights, provided an extensive tool to study ovarian cancer tumorigenesis, chemotherapeutics, and biomarkers (26–29).

Despite its utility, questions still remain whether compromising the rodent immune system affects the tumor microenvironment. Various studies have shown that cell lines implanted in immune-compromised mice can lose their histological fidelity (30–33). Likewise, monitoring disease formation and progress is also complicated with immune-compromised mice, as disease and infection rates increase when mice are handled outside their protective environment. To combat this, a small number of syngeneic models have been developed (34, 35). However, this digression from human disease presents its own complications when translating experimental results into the clinical setting.

The location of tumor formation and its histologic fidelity to the human disease is also a concern when using xenograft models. The bursal membrane in rodents encapsulates the ovary and creates a unique microenvironment unlike the human equivalent. By acting as a barrier to the peritoneal cavity, the bursal membrane could hinder the development of these tumors (36). In addition, the anatomy of the murine reproductive system departs from that of humans and contains a bicornuate uterus with the fallopian tubes embedded in the aforementioned bursa. Furthermore, the intermittent ovulatory cycle of the mouse corresponds to its rare development of spontaneous ovarian carcinomas (37).

Even with these limitations, xenograft models are still important in translational research and have broad utility. All drug treatments must show promise in animal studies prior to investigation in human clinical trials. In addition, because a high priority has been placed on characterizing the early events of HG-SOC, xenograft models can be effectively paired with *in vitro* transformation studies to characterize preneoplastic and metastatic events (Figure 3). Karst et al. demonstrated this by confirming the transformative and metastatic potential of fallopian tube secretory epithelial cells (FT-SECs) in nude mice (38). Considering this versatility and practicality, the future use of xenograft models in ovarian cancer research is a certainty.

### Cell culture models

#### OSE models

Prior to 1981, the isolation of untransformed primary ovarian tissue within the laboratory was unprecedented, making it difficult to discern molecular events related to transformation. This changed in 1981 when Adams and Auersperg isolated and transformed rat OSE (ROSE) cells with the Kirsten murine sarcoma virus (Ki-MSV) (39). The impact of this initial study led to the optimization of cell culture techniques (40, 41) and prompted investigators to start creating a vast cell bank for future studies.

Investigators took advantage of this new technology in the early 1990s when a series of studies simulated incessant ovulation through repeated *in vitro* passaging of rodent OSE cells. Investigators found that primary ROSE and mouse OSE (MOSE) cells that had undergone serial propagation exhibited increased proliferative and tumorigenic properties (35, 42, 43). Further analyses indicated that these transformed cell lines displayed similar proliferative and genomic patterns observed in human tumors. This was the first comparative analysis between a transformed cell line and its primary parental line and provided supporting evidence for the Fathalla Hypothesis.

While these studies were limited to rodent OSE cells, studies involving isolated human OSE (HOSE) cells were also being attempted (44–46). However, unlike rodent OSE cells, HOSE cells have a limited growth potential *in vitro* and require genetic perturbations to increase cellular lifespan (Figure 3). In order to achieve immortalization, two important questions require constant attention; what are the pathways critical to immortalization and how can one alter those pathways without disrupting the normal function of the cell? Initially, these genetic perturbations were achieved via retroviral transduction of either the human papilloma virus E6/E7 oncogenes or the simian virus 40 T antigen (*SV40-TAg*) (46, 47). Cell lines generated through this method displayed increased proliferation without tumorigenicity and remained proliferative after multiple passages (46, 47). Additional retroviral constructs targeting *hTERT*, *TP53*, and *RB* have all been shown to be successful in the immortalization of primary HOSE cells (48–52).

The recent development of small interfering RNAs (siRNAs) has had an impact on ovarian cancer research as well. Primarily used to silence genes through the RNA interference pathway (RNAi), Yang et al. used siRNAs to immortalize OSE cells by targeting *p53* (53) and *Rb* (54), while others have used siRNAs to explore the roles of *PTTG* (55), *CD44* (56), and *STAT3* (57). Certain investigators have even looked into siRNAs as a therapeutic agent. Huang et al. showed that by using a lipidoid-mediated delivery of siRNAs targeting *CLDN3*, OVCAR-3 xenografts showed reduced proliferation, metastasis, and tumor growth (58). The benefits of siRNAs include ease of application and more rapid results. However, specificity and cell toxicity have been a concern.

#### Fallopian tube models

The first fallopian tube epithelial cell (FTEC) culture system, developed for the purpose of studying the susceptibility of this epithelium to neoplastic transformation, was described in 2010 (59). Unlike traditional two-dimensional (2D) submerged cultures, this “*ex vivo*” system allows FTECs to grow at the air-surface liquid interface (Figure 3). This in-turn preserves the natural orientation, architecture, polarity, extracellular features, and biological functions of *in vivo* FTECs, including the retention of ciliated and secretory cells (59). Considering these advantages, this model is ideal to explore the stresses of hormone exposure, ovulation, and inflammatory response. In fact, Levanon et al. reported that in response to DNA damage the FT-SECs display delayed DNA repair kinetics compared to their ciliated cell neighbors (59). This makes secretory cells more sensitive to DNA damage and could explain why FT-SEC are susceptible to neoplastic transformation, especially in the absence of key DNA repair proteins like BRCA1 or BRCA2 (9). Despite the strengths of this model, it has two major limitations. First, it is limited by the dependence on fresh primary FT tissue. Second, the *ex vivo* cultures cannot be further propagated in culture. While they remain viable for weeks, they are not a renewable resource.

To alleviate the need for fresh tissue samples, and to create a long term self-propagating cell population, Karst et al. utilized fresh fallopian tube samples to create the first FT-SEC line (38). By transducing *hTERT* and either *SV40*-*TAg* or an shRNA targeting *p53* and mutant *CDK4^R24C^*, FT-SECs were able to overcome senescence and apoptosis (38). Further transduction of either *HRAS* or an shRNA targeting the B56γ subunit of protein phosphatase 2A (*PP2A-B56*γ) and *c-Myc* resulted in an increase in proliferation, anchorage independent growth, and tumor formation in implanted nude mice (38).

Jazaeri and colleagues reported similar results by administering an oncogenic retroviral cocktail containing a myriad of known oncogenes to primary FT-SEC (60). After a period positive selection due to proliferative advantages, the genetic profile of transformed FT-SECs was determined. Increased *c-Myc*, *HRAS*, *hTERT*, and *SV40*-*TAg* transgene expression and protein accumulation was observed. Further experimentation showed that *hTERT* and *SV40*-*TAg* expression was sufficient to overcome senescence without tumor formation in nude mice (60). This confirmed the findings of Karst et al. showing that FT-SECs are a possible source for HG-SOC.

Further confirmation of these initial results was reported by Shan et al. These investigators immortalized human FT-SECs by overexpressing *hTERT* and *SV40-TAg* (61). However, when they transduced the cells with oncogenic *HRAS* and implanted them into nude mice, they observed tumor formation that resembled poorly differentiated mucinous adenocarcinomas rather than HG-SOC (61). This is consistent with recent reports showing that Type I, low-grade tumors can emerge from the fallopian tube as well (62, 63).

Recently, FTEC models have even stepped outside traditional human cultures and expanded to baboons and pigs. A recent study used baboon FTECs immortalized with *SV40-TAg* to study the effect of ovulation on FTEC proliferation (64). Likewise, porcine oviductal epithelial cells were used to optimize *in vitro* cell culture conditions to maintain *de novo* FTEC morphological features, i.e., secretory and ciliated cells (65). These new methods could prove useful as investigations into the FTEC continue to increase.

#### Conditionally reprogramed cells

An alternative to transgene immortalization is a newly developed technique where epithelial cells are “reprogramed” into a stem cell state through conditioned media. Schlegel et al. has been able to show that primary human prostate, liver, lung, and breast epithelial cells, when co-cultured with irradiated fibroblast feeder cells in the presence of the rho-kinase inhibitor Y-27632, can undergo unlimited expansion without senescence or apoptosis (66). This increase in cell proliferation is accompanied by the up regulation of stem cell markers and a decrease in Notch signaling (66). Even more intriguing is that this phenotype is reversible. The removal of Y-27632 and feeders results in the re-differentiation of cells accompanied with their natural polarity and orientation (66). Similarly, Ince et al. showed that human mammary epithelial cells (HMECs) are able to grow indefinitely in a serum-free, chemically defined medium termed WIT (67). The optimization of these techniques for either OSE cells or FT cells would be ideal and may eliminate transgene manipulations and reduce potential off-target effects.

#### Genome engineering

Despite its successes, certain drawbacks to retroviral transduction and RNAi systems, like oncogenic effects, toxicity, and off-target effects, have prompted investigators to develop targeted genome editing systems. The application of custom DNA-binding proteins, like transcription activator-like effector nuclease (TALENs) and the clustered regularly interspaced short palindromic repeats (CRISPR)/cas genome editing systems, have produced a flurry of papers within the last few years. TALENs use a restriction enzyme engineered to recognize specific DNA sequences through the fusion of a TAL effector DNA-binding domain (68). Once a gene is targeted, double strand breaks (DSBs) are introduced and non-homologous end joining occurs (68). However, TALENs are expensive to develop and suffer from off-target effects. The CRISPR/cas system provides a cheaper alternative and works in a similar manner. By utilizing endonucleases that use dual-RNAs for site-specific DNA cleavage, investigators are able to exploit the CRISPR/cas system for RNA-programmable genome editing (69). This had been shown to be very effective and site-specific in controlling gene expression and introducing genetic mutations (70). Overall, despite its lack of validation and limited use with ovarian cancer models, promising results should spark interest and new avenues of investigation.

#### 3D cultures

In addition to conventional 2D culture systems, three-dimensional (3D) culture systems have become increasingly common. There are five major types of 3D culture systems: scaffold free for spheroid growth, scaffolds, gels, bioreactors, and microchips. In these settings, investigators concentrate on creating a more realistic environment where cells of interest can interact with surrounding tissues (71, 72). This seems relevant as studies show that differences in chemosensitivity, cell invasion, and protein expression exist when epithelial ovarian cancer cells are cultured in either 2D or 3D conditions (73–76). Difficulties associated with these models include cell removal, gelling variations, cost, and commercial availability, although the further optimization of these techniques should yield a host of useful tools.

### Animal models of ovarian cancer

#### Genetically engineered mouse models

In contrast to cell culture platforms, which rely on an artificial environment, genetically engineered mouse models are an efficient alternative for genetic modification and tumor observations *in vivo*. This is important, as questions regarding the identity of cell lines and the selective pressures of cell culture systems continue to surface (77, 78). In addition, investigators have a broad range of techniques to introduce genetic alterations in a temporal or spatial-dependent manner. These methodologies employ transgenic elements, RNAi technologies, and viruses to create both loss of function and gain of function traits within mice.

Limitations to these models include random integration of transgenic elements, limited tissue specific promoters, and difficulties achieving both spatial and temporal control simultaneously. In addition to experimental difficulties, it is also challenging to accurately mimic the human disease in rodents. For example, mice require fewer genetic alterations for tumor induction compared to humans (79–81). Furthermore, rodent tumors that are produced from defined genetic mutations do not always resemble their human counterparts (79–81). The *HRAS* oncogene is a prime example of this anomaly. Hamad et al. showed that the mechanisms of Ras-induced transformation in mice differ when compared to the mechanism of Ras-induced transformation in humans (79). By systematically comparing the murine and mammalian transformation pathways investigators highlighted a critical disadvantage to non-human model systems; the genetic and molecular disconnect between animal models and human disease. However, the ability to validate gene function and test novel therapeutics in a relevant microenvironment, when paired with relevant human studies, still makes these models especially useful (82).

#### Mouse OSE

Like other model systems, the initial ovarian cancer mouse models focused on the OSE (5, 83–85). The first ovarian cancer transgenic mouse model was developed in 2002 (86). By inducing the expression of the avian tumor virus receptor A (TVA) through the control of the *keratin-5* promoter, these investigators were able to create a cell population within the mouse that was vulnerable to avian retrovirus infection (86). However, the transient expression of *keratin-5* required OSE viral infection to occur *in vitro* with subsequent transplantation. Despite this drawback, infection with different combinations of *c-Myc*, *AKT*, and *KRas*, produced tumors in OSE cells harvested from *TVAp53*^−/−^mice and provided the first successful transgenic analysis of ovarian cancer in mice (86).

A more specific promoter, the Mullerian Inhibitory Substance Type II Receptor (MISIIR), was later identified and used by Connolly et al. to drive gynecological tissue specific transgene expression of *SV40*-*TAg* in mice resulting in the formation of ovarian carcinoma in 50% of the transgenic founders (87). However, aggressive tumor formation prohibited the study of early stage tumors and prevented reproduction. Additional studies utilizing the MISIIR promoter explored the oncogenic properties of *PTTG* and *PIK3CA*, however both had difficulties producing tumors (88, 89).

Rather than identify a specific promoter, some investigators have employed the Cre-loxP method to deliver specific genetic alterations (90). Administration of the Cre recombinase can be achieved either through injection of a viral vector (AdCre) or by crossing with a mouse generated to express the protein. This model is a clever way to circumvent problems inherent to typical transgenic models and has been used study *TP53* and *Rb* (91), *KRas* and *PTEN* (92), *PTEN* and *APC* (93), and *BRCA1* (94) within the context of ovarian cancer.

Most recently, Flesken-Nikitin and colleagues applied the AdCre system to perturb *p53* and *Rb* in a stem cell niche in the transitional zone of the bursal cavity of mice. With *p53* and *Rb* inactivated, these stem cells in the hilum region showed the earliest signs of transformation (95). However, perhaps the more interesting aspect of this study was the reporter mouse developed to characterize the fate of the hilum stem cells. A stem cell marker (*LRG5*), specific to the hilum region, was used to drive specific expression of Cre*ERT2*. In turn, subsequent tamoxifen (TAM) administration created a traceable knocked-in fluorescent probe. Results indicated that hilum cells do have the potential to repopulate the ovarian surface and suggest that stem cell niches could contribute to HG-SOC (95). Whether the hilar cells are OSE cells, or a different cell type altogether, remains to be seen. It is also worth noting that, since there is no bursa surrounding the human ovaries, it is not clear whether there is an equivalent structure or cell type in humans.

Overall, the mouse models developed thus far have focused on the OSE and some have exhibited difficulties with tumorigenicity, female reproduction, anatomical anomalies, and transient expression. Likewise, while these models have offered insight into genes that are important to transformation, they have not provided insight into HG-SOC preneoplastic lesions as such lesions have yet to be identified in the ovary. We anticipate that animal models that target the fallopian tube secretory cell will provide additional insights.

#### Mouse fallopian tube

The first mouse model targeting the extra-ovarian Mullerian epithelium was developed by Miyoshi et al. By exploiting the promoter of the murine oviduct-specific glycoprotein, Miyoshi was able to drive expression of the *SV40*-*TAg* in the oviduct, uterus, vagina, and ovary. Except for the ovary, subsequent tumor formation throughout the female reproductive tract was observed (96). Tumor formation was reduced in ovariectomized mice, but when estradiol was injected subcutaneously a dramatic increase in hyperplasia of the extra-ovarian Mullerian epithelia was observed (96). This suggests that ovarian cancer could originate outside the ovary, and that these preneoplastic lesions are highly reliant on hormone regulation pathways involving the ovary.

More recently, Kim et al. disabled *DICER* and *PTEN* using the anti-Mullerian hormone receptor type 2-directed Cre (*Amhr2*-Cre) (97). HG-SOC with aggressive metastasis was observed in these mice resulting in 100% death. In addition, the fallopian tube displayed the earliest lesions and cancer was prevented when the fallopian tube was removed at an early age (97). However, the first signs of increased proliferation within the fallopian tube appear to reside in the stromal compartment, counterintuitive to the epithelial properties presented in the advanced HG-SOC. Equally vexing was the low *p53* expression in mouse tumors, a protein known to be mutated and highly expressed in almost 100% of human tumors (7).

#### Other animal models

An alternative to the mouse model, which has dominated the field since its initial use, is the domestic laying hen. The hen is the only animal identified to spontaneously develop HG-SOC that is histologically and morphologically similar to human HG-SOC (98). Likewise, because ovarian cancer of the hen presents so many similarities to human ovarian cancer, there are many opportunities to explore early preneoplastic lesions, chemopreventive trials, and perform genomic analyses (99). Disadvantages include a lack of reagents and genetic manipulation technologies that target the hen, as well as anatomical discrepancies (99).

## Conclusion

Our understanding of ovarian cancer has dramatically changed in the last 10 years. In our search for a cell of origin, our evolving knowledge about the pathogenesis of the disease has led us to sites neighboring the ovary. At the same time, we now appreciate that this is a heterogeneous disease with a complex genomic landscape. In particular, HG-SOCs are marked by surprisingly few recurrent somatic mutations. Instead, this tumor exhibits a complex genome marked by copy number alterations so widespread that few other cancer types mirror its complexity. The challenge now is to elucidate the key alterations related to tumorigenesis, tumor viability, and chemotherapy resistance. In order to achieve this goal, experimental model systems must take center stage and continue to evolve to meet the demanding needs of the scientific community.

## Conflict of Interest Statement

The authors declare that the research was conducted in the absence of any commercial or financial relationships that could be construed as a potential conflict of interest.
